# Thalamic abscess in a patient with hereditary hemorrhagic telangiectasia successfully treated with an empiric antibiotic regime: case report and review of the literature

**DOI:** 10.1186/s12879-021-05955-6

**Published:** 2021-03-19

**Authors:** Xavier A. Santander, Anwar Saab, Juan Manuel Revuelta-Barbero, Elena Múñez

**Affiliations:** 1 Department of Neurosurgery, University Hospital Puerta de Hierro Majadahonda, Madrid, Spain; 2 Department of Neurosurgery, University Hospital Clínico San Carlos, Madrid, Spain; 3 Department of Infectious Diseases, University Hospital Puerta de Hierro Majadahonda, Madrid, Spain

**Keywords:** Thalamic, Abscess, Telangiectasia, Antibiotics, Case-report

## Abstract

**Background:**

Hereditary hemorrhagic telangiectasia (HHT) is a rare autosomal dominant disease associated with neurological complications, including cerebral abscesses (CA). They tend to be unique, supratentorial and lobar. While the surgical intervention is a rule of thumb when treating and diagnosing the etiology of these lesions, this is not always possible due to dangerous or inaccessible locations. We report the case of a patient solely treated with empiric antibiotics without stereotaxic intervention and satisfactory results.

**Case presentation:**

We present the case of a 21-year-old patient with a right thalamic abscess due to HHT and pulmonary arteriovenous malformations, previously embolized, treated solely with antibiotics. At first, we contemplated the possibility of a stereotaxic biopsy, but the high-risk location and the fact that our patient received a previous full course of antibiotic treatment (in another center), made us discard this intervention because of the low diagnostic yield. We started an empiric antibiotic regime. We followed up very closely the clinical and radiological evaluation the next weeks, adjusting our antibiotic treatment when necessary. The results were favorable from both the radiological and clinical aspects and 6 months after the diagnosis the images show its almost complete disappearance.

**Conclusion:**

Carefully tailored antibiotic-only regime and vigilance of its adverse effects and close radiological following is a good treatment approach when surgery is not an option.

## Background

Osler-Weber-Rendu syndrome or hereditary hemorrhagic telangiectasia (HHT) is a rare entity consisting of angiomata of the skin, mucous membranes and viscera. It is an autosomal dominant disorder with high penetrance [1, 2]. From the genetic point of view, HHT is a heterogeneous disorder. At least three genes have been identified as culprits: Endoglin, activin A receptor type II-like 1 and SMA-and MAD related protein 4 [3]. Common features are diverse types of bleeding originated from the angiomas, including epistaxis, hemoptysis, hematuria and melaena. The diagnosis is based on the Curacao criteria [4].

Mortality and morbidity are related to central nervous system (CNS) complications, like cerebral abscess (CA), ischemic stroke, transient cerebral ischemic attack, and cerebral hemorrhage due to arterio-venous malformations. In the presence of pulmonary arterio-venous malformations (P-AVM) there is a well-stablished relationship with CA [5].

When a CA is diagnosed, there is an accepted course of action: surgery, diagnosis and antibiotic treatment. Literature reporting cases solely treated with antibiotics without surgery are scarce in the literature and our case is a great example of how to get satisfactory results tailoring a personalized empiric antibiotic treatment when surgery is not doable.

## Case presentation

A 21-year-old man, with a previous diagnosis of HHT and embolization of multiple P-AVM in the past, was admitted at another hospital, while on holidays, with fever, headache and malaise. A non-enhanced computed tomography (CT) of the head showed no significant findings. He was diagnosed with typhoid fever based on clinical features and serology. Consequently, he was discharged with oral antibiotic therapy consisting of ciprofloxacin 500 mg bid and cotrimoxazole 800/160 mg bid.

**Fig. 1:**
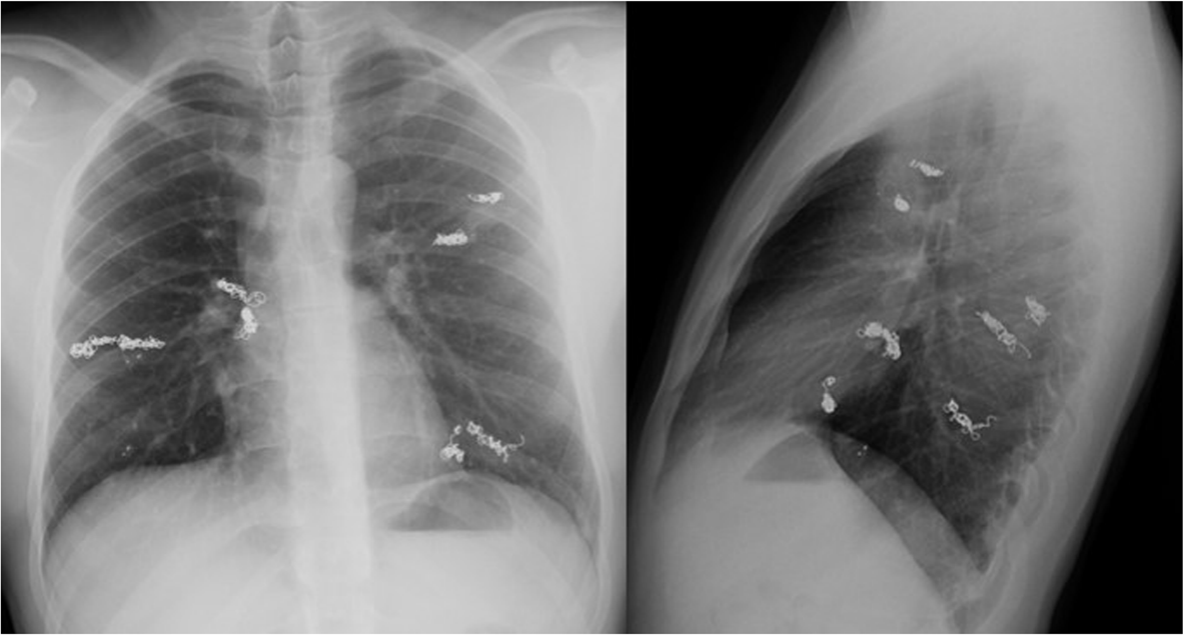
Chest X-radiograph showing previous material embolization but no signs of infection coming from the lower air pathway

Two weeks later, he came to our emergency department because of the persistent fever and headache. At our center, the neurologic, cardiac and pulmonary examination were normal. Routine hematology showed a white cell count of 17.6 × 10^3^/ml leucocytes (4.0–11.5) and 14.33 × 10^3^/ml neutrophils (1.5–7.5). The C-reactive protein was 2.00 mg/L (0.1–10) and the arterial blood gases were normal. A chest radiograph appeared to be normal other than visualization of previous embolization material (Fig. 1).

**Fig. 2:**
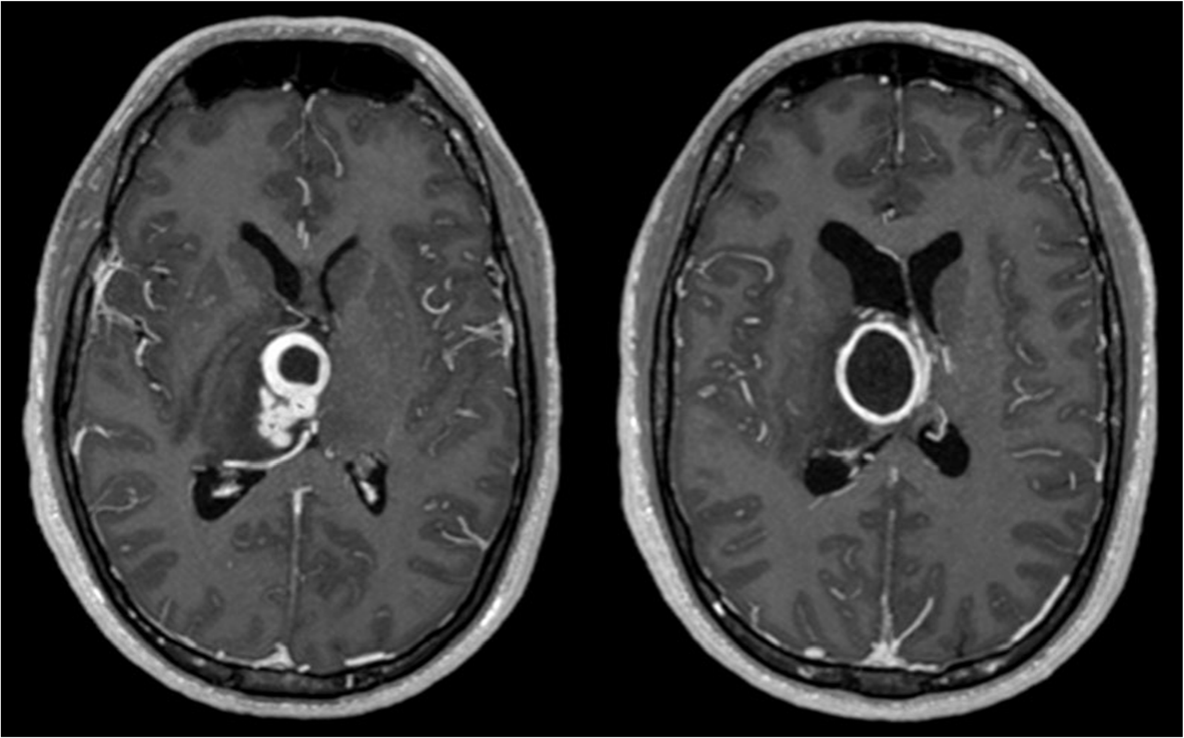
Initial T1W1 C+ MRI at the time of diagnosis. The presence of a typical ring-like enhancing region suggesting a polylobulated abscess is shown. The lesion was in the right capsular-thalamic region, deforming the lateral ventricular architecture and deviating the midline. T1W1 C+ (T1 weighted image with gadolinium enhancement)

A contrast-enhanced CT scanner of the head revealed a mass in the right thalamic-capsular region, compatible with an abscess. An MRI, performed later, confirmed the findings (Fig. 2) showing the typical ring-like contrast enhancement.

Further investigations were performed. A cardiac transesophageal echography was normal. A CT of his lungs confirmed the presence of embolization material inside the lumen of pulmonary vessels feeding the malformations. After reviewing the images with our radiology team, they concluded that the lesions were successfully treated. Pulmonary angiography was not performed in light of these results and because our patient was being followed for that pathology in another center.

## Treatment

After carefully evaluating the images and discussing treatment options with our patient, we decided to start with intravenous empiric medical treatment, due to the high risk of a surgical intervention. However, we contemplated the possibility of a stereotaxic biopsy, but considering our patient received a previous full course of antibiotic treatment we discarded this intervention because of the low diagnostic yield. Thus, treatment was started with intravenous linezolid 600 mg tid, plus ceftazidime 2 g tid and metronidazole 500 mg qid. A course of corticosteroids, dexamethasone 4 mg tid, was also initiated.

**Fig. 3:**
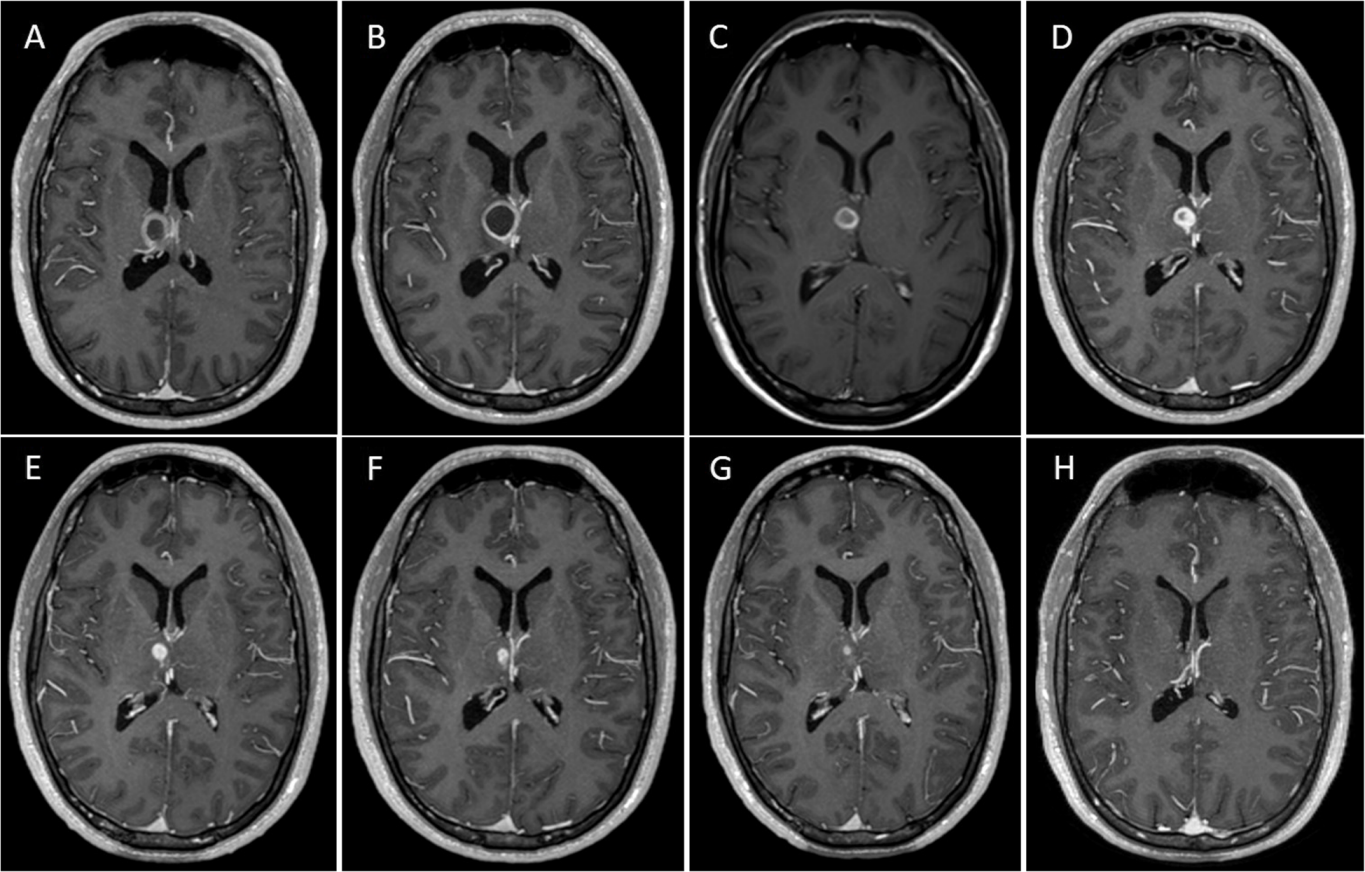
Multiple T1WI C+ sequences showing the radiological evolution of our case. **a** This first image shows the improvement after the first course of antibiotics and corticosteroids. **b** The radiological and clinical worsening, shortly after the waning of corticosteroids and the change of antibiotics. **c** Sequence obtained 10 days after the last image, showing good evolution after the reintroduction of ceftriaxone. From here on our patient had a maintained good evolution during following months, after we refined the empirical antibiotic treatment. **d** Last image showing steadiness in the size of the lesion, previous to discharge with good clinical stability. **e**-**f**-**g** Images taken at 3, 4 and 5 months after discharge. **h** The last and actual image showing no further presence of the lesion. T1W1 C+ (T1 weighted image with gadolinium enhancement)

After 2 weeks, a new CT evidenced a favorable radiological evolution with reduction of the lesion. He was afebrile, the headache had disappeared, and his general condition had dramatically improved. We switched linezolid and metronidazole to an oral regimen. After 1 week he remained asymptomatic and we decided to rotate the antibiotic regimen to ceftriaxone 2 g bid and ciprofloxacin 750 mg bid, maintaining the linezolid and metronidazole, due to its better tolerance profile and towards to a possible discharge. By that time, he was almost finishing his waning course of corticosteroids. We performed a new MRI control showing good radiological results (Fig. 3a).

Three days after, he presented headache of moderate intensity and fever. A new CT scan showed an increase in size of the thalamic abscess that we confirmed with a new MRI (Fig. 3b). We attributed this evolution to the change of antibiotics. Our patient remained neurological stable and that’s why we decided to reintroduce ceftazidime 2 g tid and re-started a new course of corticosteroids, discarding a surgical intervention. He started to improve again. A new MRI performed 10 days after this regimen demonstrated a reduction in the size of the lesion (Fig. 3c).

In the day 21 (Table 1), after a hematologic control showed an increase in hepatic enzymes: AST: 192 U/L (6.0–40) ALT: 649 U/L (6.0–40), GGT: 666 U/L (8.0–61), gastrointestinal discomfort and hyporexia, we performed an abdominal echography and found signs of hepatopathy. Then again, due to the toxicity of ceftazidime and metronidazole, we changed to a new regimen consisting of linezolid and meropenem 1 g tid. Another MRI showed further shrinking of the lesion (Fig. 3D).

**Table 1:** Hepatic profile and antibiotic regime during the treatment

Hepatic enzyme (U/L)	ANTIBIOTIC REGIME														
	LINEZOLID METRONIDAZOLE CEFTAZIDIME				CEFTRIAXONE CIPROFLOXACINE LINEZOLID METRONIDAZOLE		LINEZOLID METRONIDAZOLE CEFTAZIDIME			LINEZOLID MEROPENEM			COTRIMOXAZOLE MOXIFLOXACIN		
AST	14	15	21	35	24	36	85	130	192	161	159	97	61	35	21
ALT	22	28	24	30	42	43	189	270	649	473	314	397	224	113	41
GGT	22	26	27	33	42	51	150	225	666	610	568	358	64	38	22
**Therapy Day**	1	5	10	14	20	27	33	40	48*	51	55	60	1 m	2 m	3 m
	INPATIENT												OUTPATIENT		

*Day 48 is 21 days after initiating the third antibiotic course

He remained stable with no neurologic deficit and a clear improvement of his hepatic profile. Because of the prolonged therapy with linezolid, he presented leucopenia: 2.8 × 10^3^/ml leucocytes (4.0–11.5). After a final antibiotic rotation to cotrimoxazole and moxifloxacin orally, he was discharged with radiological stability, clinical improvement and corrected hematologic disturbances.

## Discussion and conclusion

Almost 50% of patients with P-AVM have a family history of HHT, and 10% of members of HHT families have a P-AVM [6]. As estimated by Roman [7] the frequency of a brain abscess in a patient with HHT and a P-AVM is 5%, this is × 10^3^ times the risk of developing CNS infection in the general population [6]. Although the pathogenesis is not well understood, it is believed to be the result from right-to-left pulmonary shunts and paradoxical embolization. Two main mechanisms have been proposed: 1) septic micro emboli that can reach the CNS, from the digestive tube, favored by polycythemia and hypoxic conditions that decrease resistance of cerebral tissue to bacteria invasion and 2) secondary infection of previous brain microinfarctions during transient bacteremia [8–11].

As a rule of thumb, the first diagnosis to be considered in a patient with positive history of P-AVM and HHT and a cerebral mass should be a CA. This was the case of our patient. On the other hand, one should think about P-AVM when a patient has a cerebral abscess and no previous history suggesting any infectious origin [12, 13]. Actually, many patients with P-AVM are asymptomatic before presentation with neurologic complications such as brain abscess or stroke [14].

These CAs, when secondary to HHT, are generally supratentorial (frontal lobe in 40% of cases), lobar and unique [9, 15]. Our patient had multiple satellite lesions around a main one in the thalamic region (deep basal ganglia). This presentation is rare and estimated around 4% of all CAs [15].

The gold standard for P-AVM is the digital subtraction angiography. It allows diagnosing and treating by embolizing the main vessel feeding the malformation. However, appropriate management does not necessarily exclude the possibility of CA recurrence. In the series by Mathis et al., 15.4% of patients had recurrence even when they were adequately treated [15]. The recurrence in other locations, different from the first presentation, reveals the crucial role of P-AVM. Failure of treatment after a successful embolization of the fistula is considered low by 5 years as reported by White et al. [16].

The treatment of every CA should be multidisciplinary. It must encompass a medical and surgical approach. General recommendations are: 1) surgical drainage if lesion > 2.5 cm in diameter; 2) CT or MRI imaging every 15–20 days and 3) 6–8 weeks of intravenous antibiotic treatment [9]. The concept of treating only with antibiotics when the lesion is not amenable to surgery is not wild; however, literature describing this type of approach is scarce. This is why we believe this case can highlight the importance of taking into account this kind of approach. Non-operative treatment of CA has been successfully reported in some cases but this option should be reserved for poor surgical candidates or small lesions in inaccessible areas [9, 17]. In the series reviewed by Sell et al. [9], one patient had no biopsy and survived only with medical treatment. Nonetheless, stereotactic drainage is usually considered the treatment of choice [18]. In our case our patient had a thalamic CA, from our point of view, not amenable to surgery and he received previously a course of antibiotics at another center. We considered a surgical intervention not recommended because of the high risks and low yield ratio of diagnosis after antibiotics treatment. Thus, we decided to manage this CA only with antibiotics.

We agree there is a limitation when using antibiotics if no bacterial agent is previously identified, but the vast majority of microorganisms found in CA related to HHT involves anaerobic or facultative anaerobic bacteria. Streptococcus is the most common organism [6]. Staphylococcal infection, although a common organism in extra-cerebral infections in this group, is somewhat extraordinary in the CNS, being more common (up to 30% and always associated with endocarditis) in patients with CA and no HHT. Moreover, CA related to HHT tend to be of multiple germs (2–3 bacteria) which is a striking difference when compared to non-HHT related CA [15]. This highlights the utmost importance of carefully selecting and tailoring the empiric antibiotic therapy, when there is no option to obtain samples for cultures. Mortality, historically considered high in the pre-antibiotic era, has considerably improved in part due to the advancements in imaging modalities, less invasive surgical techniques and a broader antibiotic spectrum and efficacy.

As of 6 months after the initial diagnosis, our patient is asymptomatic and his lesion has almost completely disappeared as demonstrated in the radiological follow-up (Fig. 3 e, f, g and h).

Proper knowledge of the relationship between CA and HHT is vital to raise the level of suspicion, especially in patients with no underlying cause. Previous detection and embolization of P-AVM reduces the recurrence of CA but does not exclude them completely, as was the case of our patient.

Finally, our case highlights that carefully selecting the antibiotic regime, vigilance of adverse effects and close radiological following is of utmost importance when surgery is not an option.

## Data Availability

Our manuscript is a case-report. The available-data section is not applicable.

## Abbreviations

ALT: Alanine transaminase
AST: Aspartate transaminase
Bid: Twice per day
CA: Cerebral abscess
CNS: Central Nervous System
CT: Computed Tomography
GGT: Gamma-glutamyl transpeptidase
HHT: Hereditary Hemorrhagic Telangiectasia
MRI: Magnetic Resonance Image
P-AVM: Pulmonary Arteriovenous Malformation
Qid: Four times a day
T1W1: T1 Weighted image
Tid: Thrice per day
